# Congenital Cytomegalovirus Infections Mother-Newborn Pair Study in Southern Ethiopia

**DOI:** 10.1155/2021/4646743

**Published:** 2021-12-30

**Authors:** Mengistu Hailemariam Zenebe, Zeleke Mekonnen, Eskindir Loha, Elizaveta Padalko

**Affiliations:** ^1^School of Medical Laboratory Sciences, Hawassa University College of Medicine and Health Sciences, Hawassa, Ethiopia; ^2^School of Medical Laboratory Sciences, Jimma University Institute of Health, Jimma University, Jimma, Ethiopia; ^3^Department of Diagnostic Sciences, Ghent University, Ghent, Belgium; ^4^Centre for International Health, University of Bergen, Bergen, Norway; ^5^Chr. Michelsen Institute, Bergen, Norway; ^6^Laboratory of Medical Microbiology, Ghent University Hospital, Ghent, Belgium

## Abstract

**Introduction:**

Congenital cytomegalovirus (cCMV) is a common cause of neurodevelopmental delays and sensorineural hearing loss of infants, yet the prevalence of cCMV and the associated factors in Ethiopia are not studied. Hence, this study was to assess the prevalence and associated factors of cCMV in Southern Ethiopia. *Methodology*. A mother-newborn pair cross-sectional study was conducted at Hawassa University Comprehensive and Specialized Hospital, Ethiopia. Newborn's saliva sample was tested for cCMV using Alethia CMV molecular assay. Mothers' serum was tested serologically for anti-CMV IgM and IgG by EUROIMMUN ELISA. Pregnant women responded to a questionnaire about their previous and current obstetric history and sociodemographic characteristics. The chi-square (*χ*^2^) test and independent-sample *t*-test were used to determine the associations between infections and possible risk factors; then, potential variables were screened for multivariable analysis.

**Results:**

A total of 593 mother-newborn pairs were assessed. CMV was detected in 14 of 593 newborn saliva swabs (2.4%; 95% CI 1.2–3.7). As assessed by CMV IgM-positive results, maternal CMV seropositivity was 8.3% (49/593); thus, the rate of mother-to-child transmission of CMV was 28% (14/49) among CMV IgM-positive women. Congenital CMV infection was significantly associated with maternal exposure through nursery school children in the household, women sharing a feeding cup with children, and any of the detected curable STIs during pregnancy. Birth weight was negatively associated with CMV infection. Maternal age, gravidity, level of education, and sharing of children feeding utensils were not associated with cCMV infection.

**Conclusion:**

A high rate of cCMV infection in the absence of awareness demands further in-depth investigation in Ethiopia. Thus, policymakers must take appropriate action through the antenatal care system for prevention strategies and put in place a constant health education and awareness creation of pregnant women about the causes of infection and hygienic measures.

## 1. Introduction

Cytomegalovirus (CMV) is the most common congenital viral infection passed from pregnant women to babies during pregnancy [1]. Congenital CMV (cCMV) is a leading cause of neurodevelopmental disabilities, including mental retardation, sensorineural hearing loss, and vision impairment [2]. CMV intrauterine transmission follows in both maternal primary infection and nonprimary infection that could be either reinfection with a different CMV strain or reactivation of previous infection [3]. Reactivation can be determined by a variety of viral, environmental, and host factors mainly coexisting with sexually transmitted infections (STI) including HIV [4].

It is known from the available scientific literature [5–8] that STI during pregnancy can increase the reactivation or reinfection rate of maternal CMV infection. Consequently, there will be an expected rise in the sexual transmission of CMV. However, there are not a lot of studies available to address this item more in the depth. Additionally, whether the coinfection during pregnancy increases congenital transmission is not well studied.

Unlike congenital rubella, the maternal immunity acquired against CMV prior to conception does not confer complete protection to the developing fetus [9]. Therefore, a significant rate of cCMV was reported in places where higher rates of maternal seroprevalence were reported. The prevalence of cCMV varies across geographical regions with a lower prevalence in developed countries as compared to Africa, South Asia, and South America [10]. On the other hand, cCMV remains underestimated as a healthcare problem in the entire world while being the main congenital infection. The live birth prevalence of cCMV in developed countries is as low as 0.6 to 0.7% but higher (1–5%) in developing countries [11], where some African countries even reported higher than pooled average like 5.7% in Egypt [12] and 5.9% in South Africa [13].

Despite its burden, cCMV infection often goes undetected at birth because the majority of affected infants are asymptomatic or present with symptoms that are sufficiently nonspecific that do not prompt clinicians to suspect CMV infection. Screening programs, both in pregnant women and in newborns, have not been developed and implemented. Therefore, conducting prevalence cCMV at birth in Ethiopia is important to know the country level burden of the problem and for possible prevention measures. This study is the first in Ethiopia performed on newborn saliva samples. A previous study in Ethiopia conducted using the serological test on cord blood had reported 1.3% of cCMV [14]. The recommended diagnosis of newborn CMV infection is by molecular assay of either urine or saliva samples collected within the first 2-3 weeks after birth [15]. In this study, we used Alethia CMV molecular assay for CMV testing using neonatal saliva [16].

The study aimed to determine the prevalence of cCMV among live births in parallel to the serostatus of their mothers for CMV IgM and IgG. The study also identifies the associating factor and possible efforts required in the prevention of maternal infection to reduce the burden of congenital CMV disease.

## 2. Methods

### 2.1. Study Area and Design

A cross-sectional study was conducted on consecutive pregnant women who came for delivery and agreed to participate with their newborns at Hawassa University Comprehensive and Specialized Hospital (HU-CSH) obstetric ward from August to October 2020. The HU-CSH is one of the teaching hospitals serving as a referral center for both public and private hospitals for more than 5 million inhabitants in the southern region and the neighboring region of Ethiopia. The hospital has 500 beds, accommodating around 2,500 pregnant women for antenatal care (ANC) visits and conducting about 5,400 deliveries annually.

### 2.2. Study Period and Enrollment

Participants were enrolled at the obstetric ward at a time when they came for delivery after obtaining their full consent from August to October 2020. Among the enrolled 600 pregnant women, a total of 608 infants were born (8 twin), of which 11 were stillbirths, 3 were early neonatal deaths, and one was unable to give a sample due to ICU admission. Therefore, 593 neonates were enrolled for assessment of cCMV infection. The pregnant women were screened for CMV IgM and IgG antibodies. Sampling was based on convenience and continued until the intended sample size was reached. Nonresponse during data collection was solved by taking subsequent participants.

### 2.3. Study Variables

The dependent variable was newborns' saliva test results for cCMV. The independent variables were sociodemographic characteristics, obstetric history of the mother, and the newborns' characteristics like birth weight and sex.

### 2.4. Sociodemographic, Obstetric, and Behavioral Data

The trained midwife at the obstetric ward provided general information about the study to pregnant women who came for delivery. Pregnant women agreeing to join in the study were interviewed using a structured questionnaire translated in Amharic, the language spoken by most people in the study area. The questionnaire was piloted on random pregnant women not included in this study at the same hospital before data collection to ensure the validity and feasibility of the questions. Information related to sociodemographic characteristics (e.g., age, marital status, and educational level), obstetric history, and behavioral data was collected. Newborn's birth weight and sex were recorded from medical records. According to the World Health Organization (WHO), low birth weight is defined as weight at birth of less than 2,500 g, while preterm is babies born alive before 37 weeks of pregnancy [17].

### 2.5. Sample Collection and Storage

The midwife-nurse drew a 3 ml blood specimen from each pregnant woman before birth under aseptic condition and considered all current COVID-19 prevention guidelines. Blood samples were processed, and serums were stored at −20°C until analysis for CMV. At the same time interrelated with the first phase of this project, vaginal swab specimen was also collected for the first 350 mothers using Xpert CT/NG Vaginal/Endocervical Specimen Collection Kit (Cepheid, Sunnyvale, California, USA) for curable STI diagnosis [18].

The saliva swabs were collected using nylon-flocked swabs (Copan Italia, Brescia, Italy) according to the instruction guidelines set by Alethia CMV molecular assay at least one hour after the consumption of breast milk [19]. Immediately after collection, samples were shipped to the microbiology laboratory and stored at −20°C until analysis.

### 2.6. Laboratory Methods

The saliva swab samples were analyzed using the Alethia CMV molecular assay. All laboratory procedures were carried out in accordance with appropriate guidelines and regulations set by the manufacturer [19]. Before the actual assay procedure, the frozen samples were incubated at 19–30°C for 2-3 minutes.

Serologic test of mother's serum was performed using a commercially available enzyme immunoassay (ELISA) kit (EUROIMMUN Medizinische Labordiagnostika AG, Lübeck, Germany) for anti-CMV IgG and IgM according to the manufacturer's instructions.

### 2.7. Data Analysis

Statistical analysis was conducted using SPSS software (version 20.0). The chi-square (*χ*^2^) test and independent-sample *t*-test were used to determine associations between infections and possible risk factors and screen potential variables for multivariable analysis. All explanatory variables with a *p* value less than or equal to 0.2 in the bivariate analysis were included in the multivariable logistic regression model to identify variables that were associated independently. Odds ratios with 95% confidence intervals (CIs) were reported, and a result was considered statistically significant at *p* < 0.05.

## 3. Results

### 3.1. Characteristics of Study Participants

Of the 593 saliva samples tested, 14 specimens (2.36%) were positive for CMV. Among the 14 detected CMV-positive newborns, 8 (2.9%) were male children. The mean birth weight was 2,614 grams for CMV-positive and 3,287 grams for CMV-negative newborns. The mean age of CMV-positive newborns' mothers was 28.0 ± (SD) 6.0 and the CMV-negative newborns' mothers was 27.1 ± (SD) 5.1. Eleven out of 14 cCMV-positive newborns were from mothers who have under-five children and nursery school baby in the household. Nearly one-fourth of the women were primigravida. About half of them have nursery school children in their household, 14% (84/593) were currently unmarried, 79% were residing in an urban setting, and 96% were unaware of congenital transmitted infection (Table 1).

### 3.2. Maternal CMV Seroprevalence and STIs

According to the CMV serology of mothers, 8.3% (49/593) and 89.7% (532/593) were seropositive for CMV IgM and CMV IgG, respectively. All CMV IgM-positive women were also positive for IgG. About 10% of mothers tested negative for both anti-CMV IgG and IgM. Among 348 mothers tested for STI, 49 were positive for any of curable STIs (*C. trachomatis*, *N. gonorrhoeae*, and *T. vaginalis*) (Table 2).

### 3.3. Birth Prevalence of Congenital CMV

Among 593 participant infants, 14 had a positive saliva test for CMV giving a prevalence of 2.36% (95% CI, 1.2%–3.7%). All CMV-positive newborns' mothers were seropositive for both IgM and IgG antibodies. In this study, the cCMV transmission rate among CMV IgM-positive mothers was 28% (14/49). On the other hand, among the 49 STI-positive mothers, 4 (8.2%) newborns were tested positive for cCMV (Table 2).

### 3.4. Associated Factors for Congenital CMV Infections

In bivariable analysis, a fivefold cCMV infection was documented among women having nursery school baby in the household compared with not having nursery school baby, and a fourfold cCMV infection was documented among women employed as daycare workers compared with women not working in daycare. In multivariable logistic analysis, cCMV infection was associated with households having nursery school children (AOR = 5.7, 95% CI: 1.0–31.8) and women sharing a feeding cup with children (AOR = 5.3, 95% CI: 1.1–26.8). While birth weight was negatively associated with CMV infection though the effect was minimal, that is, for a 1,000 grams increase in birth weight, the odds of CMV infection were decreased by 4% (AOR = 0.996, 95% CI: 0.994–0.999). Furthermore, after adjusting for STIs, the observed associations of birth weight were somehow consistent and the cCMV infection showed association with mothers positive for STIs (AOR = 9.2, 95% CI: 1.8–47.6) (Table 3).

## 4. Discussion

The study assessed the prevalence of cCMV among newborns in Southern Ethiopia, which is the first document about cCMV prevalence in Ethiopia.

The high level (2.4%) of cCMV among newborns presented in this study is in agreement with the previous reports in Africa from South Africa (2.5%) [20], (2.9%) [21], and Kenya (3.6%) [22]. Likewise, our finding is in line with the systematic reviews that reported a range of 0.6–6.1% for developing countries [23, 24]. Our result is higher compared to previous studies in Egypt (1.3%) [25] and Ivory Coast (1.4%) [26]. However, compared to our finding, a higher cCMV prevalence was reported from other studies from Egypt (5.7%) [12], Nigeria 3.8% [27], Gambia (5.4%) [28], (3.9%) [29], Zambia (3.8%) [30], South Africa (5.96%) [13], and Mozambique (6.3%) [31].

Transplacental transmission of CMV is approximately 35% of pregnancies in which a maternal primary infection occurs. Transplacental transmission rates are higher if women get infected in the third trimester even if the rates of transmission vary depending on the characteristics of the maternal population. However, the transmission rate of nonprimary maternal CMV infection is minimal estimated from 1 to 2% [9, 32]. In this study, the mother-to-child transmission rate of cCMV was 28% among CMV IgM-positive mothers, which is comparable with the previous findings [9, 33, 34]. In our setup, it was not possible to differentiate the type of infection as primary or secondary. Therefore, in all cases an appropriate prevention strategy including awareness conception to healthcare professionals and pregnant women during their antenatal care might reduce the rate of maternal and cCMV infections.

The high rate of cCMV and maternal CMV seropositivity, in the presence of prior maternal immunity to CMV observed across several African studies [12, 13, 22, 31], indicates the presence of a lot of factors implicating reinfection or reactivation root during pregnancy. Up to now, the reported possible predictors for maternal CMV infection and subsequent congenital transmissions to their newborn were sexual contact during pregnancy, being multigravida, being daycare workers and caring for young children, lower educational level, and having children who attend daycare centers in the household [7, 33].

Our study also reported a significant association of cCMV infection with maternal exposure through nursery school children in household and mother employed at the child daycare center. Nursery school children easily get infected in school and frequently shed CMV in their saliva or urine for many years continuously, and in turn CMV readily spreads in preschool setting [35]. Thus, pregnant women having a young child in daycare are at increased risk of CMV reinfection or seroconversion [36, 37]. It would be considered more risk due to the high possibility of direct contact with contagious secretions from these children essentially in a situation of poor hygienic practice like in Ethiopia [38]. Likewise, pregnant women working in child daycare could also be at high risk due to close contact with a possibly infected child and their contagious secretions.

Congenital CMV infection has been proposed as a risk factor for low birth weight [39]. This study confirmed the minimal association of birth weight with newborn CMV infection. In fact, a lot of other factors implicate low birth weight [40], which we did not assess in its fullest spectrum, and congenital CMV infection might be important.

Likewise, newborn cCMV infection was found to be significantly associated with mother CMV coinfected with one or more of curable STIs detected at delivery. It increased the risk about a fivefold likelihood chance to transmit cCMV to their newborn. Certainly, for STI-positive mothers, CMV infection might be reactivated or might be responsible for reinfection with different viral strains in high-seroprevalence populations, which can increase the chance of congenital transmission beyond the possible outcome of coinfections [6, 7]. Our finding is comparable with a study reported from the USA that states women with STIs were 6 times more likely to have an infant with cCMV [8, 35].

We acknowledge that we were unable to perform confirmation testing for CMV-positive newborns' saliva by urine sample as a gold standard method. The study did not assess maternal CMV serology at early pregnancy, so we were unable to differentiate maternal seropositivity for CMV IgM as either primary or secondary (reinfection or reactivation). Additionally, it was a hospital-based study so that was not representative of all newborns in the locality. Therefore, future researchers may need to consider greater sample sizes.

## 5. Conclusions

A high rate of congenital CMV and maternal CMV seropositivity demand further in-depth investigation in Ethiopia. In the absence of a diagnostic facility, policymakers have to take appropriate action through the antenatal care system for prevention strategies and educating pregnant women about the causes of infection and hygienic measures. Moreover, training and awareness conception to healthcare professionals have to be adequately addressed to deliver accurate information on consequences and preventive actions.

## Figures and Tables

**Table 1:** Characteristics of mothers and newborns with congenital CMV infection.

Characteristics	Total *N* (%)	Newborn CMV		*p* value
		Positive *N* (%)	Negative *N* (%)	
Sex of neonate				
Male	276 (46.5)	8 (2.9)	268 (97.1)	0.421
Female	317 (53.5)	6 (1.9)	311 (98.1)	
Gestational age at birth				
Term	529 (89.2)	9 (1.7)	520 (98.3)	0.006
Preterm	64 (10.8)	5 (7.8)	59 (92.2)	
Birth weight in grams: mean (SD)		2614.3 (256.7)	3287.4 (550.2)	<0.001
Maternal age (years): mean (SD)		28 (6.0)	27.1 (5.1)	0.506
Marital status				
Married	509 (85.8)	11 (2.2)	498 (97.8)	0.435
Currently not married	84 (14.2)	3 (3.6)	81 (96.4)	
Residence				
Urban	470 (79)	12 (2.6)	458 (97.4)	0.550
Rural	123 (21)	2 (1.6)	121 (98.4)	
ANC follow-up during pregnancy				
Yes	569 (96)	13 (2.3)	556 (97.7)	0.558
No	24 (4)	1 (4.2)	23 (95.8)	
Employed as daycare worker				
Yes	40 (6.7)	3 (7.5)	37 (92.5)	0.040
No	553 (93.3)	11 (2.0)	542 (98)	
Employed as healthcare worker				
Yes	32 (5.4)	2 (6.3)	30 (93.7)	0.156
No	561 (94.6)	12 (2.1)	549 (97.9)	
Educational level				
Primary and below	217 (36.6)	3 (1.4)	214 (98.6)	0.244
Secondary and above	376 (63.3)	11 (2.9)	365 (97.3)	
Gravidity				
Primigravida	143 (24.1)	3 (2.1)	140 (97.9)	0.812
Multigravida	450 (75.9)	11 (2.4)	439 (97.6)	
Previous adverse pregnancy outcome^*∗*^				
Yes	38 (8.5)	1 (2.6)	37 (97.4)	0.812
No	412 (91.5)	10 (2.4)	402 (97.6)	
Knowledge on congenitally transmitted infections				
Yes	25 (4.2)	1 (4.0)	24 (96.0)	0.586
No	568 (95.8)	13 (2.3)	555 (97.7)	
Under-five children in the household				
Yes	393 (66.3)	11 (2.8)	382 (97.2)	0.332
No	200 (33.7)	3 (1.5)	197 (98.5)	
Nursery school baby in the household				
Yes	258 (43.5)	11 (4.3)	247 (95.7)	0.015
No	335 (56.5)	3 (0.9)	332 (99.1)	
Sharing a cup with children				
Yes	105 (17.7)	5 (4.8)	100 (95.2)	0.085
No	488 (82.3)	9 (1.8)	479 (98.2)	
Sharing teeth brush with children				
Yes	38 (6.3)	2 (5.3)	36 (94.7)	0.239
No	555 (93.6)	12 (2.2)	543 (97.8)	
Sharing eating utensil with children				
Yes	88 (14.8)	1 (1.1)	87 (98.9)	0.425
No	505 (85.2)	13 (2.6)	492 (97.4)	

^*∗*^Previous adverse pregnancy includes early neonatal death, stillbirth, and preterm birth (*n* = 450).

**Table 2:** Maternal STIs and CMV seropositivity with newborn congenital CMV infection.

Characteristics	Total (*N* = 593) *n* (%)	Newborn CMV-positive (*N* = 14) *n* (%)	*p* value
CMV IgM			
Positive	49 (8.3)	14 (28.6)	<0.001
Negative	544 (91.7)	0 (0)	
CMV IgG			
Positive	532 (89.7)	14 (2.6)	0.381
Negative	61 (10.3)	0 (0)	
Any curable STIs (*n* = 348)			
Positive	49 (14.1)	4 (8.2)	0.016
Negative	299 (85.9)	5 (1.7)	

**Table 3:** Unadjusted and adjusted associated factors with newborn congenital CMV infection.

Characteristics	Unadjusted		Adjusted		Adjusted^†^	
	OR 95% CI)	*p* value	OR (95% CI)	*p* value	OR (95% CI)	*p* value
Birth weight	0.997 (0.995–0.998)	<0.001	0.996 (0.995–0.998)	<0.001	0.996 (0.994–0.999)	0.003
Nursery school baby						
Yes	4.9 (1.4–17.8)	0.015	5.2 (1.3–20.5)	0.019	5.7 (1.0–31.8)	0.047
No	1					
Daycare worker						
Yes	4.0 (1.1–4.9)	0.040	3.0 (0.6–14.7)	0.180	1.8 (0.2–19.4)	0.629
No	1					
Employed as healthcare worker					LD	
Yes	3.1 (0.7–14.3)	0.156	3.9 (0.6–27.8)	0.171		
No	1					
Sharing a cup with children						
Yes	2.7 (0.9–8.1)	0.085	4.6 (1.3–17.1)	0.021	5.3 (1.1–26.8)	0.043
No	1					
Any curable STIs						
Yes	5.2 (1.4–20.2)	0.016	NA		9.2 (1.8–47.6)	0.008
No	1		NA			

^†^Adjusted for STIs; NA: not applicable; LD: category lack data.

## Data Availability

The data used to support the findings of this study are available from the corresponding author upon request.
